# Solubilization of lipids and lipid phases by the styrene–maleic acid copolymer

**DOI:** 10.1007/s00249-016-1181-7

**Published:** 2016-11-04

**Authors:** Juan J. Dominguez Pardo, Jonas M. Dörr, Aditya Iyer, Ruud C. Cox, Stefan Scheidelaar, Martijn C. Koorengevel, Vinod Subramaniam, J. Antoinette Killian

**Affiliations:** 1 Department of Chemistry, Faculty of Science, Membrane Biochemistry and Biophysics, Bijvoet Center for Biomolecular Research, Padualaan 8, 3584 Utrecht, The Netherlands; 2 Nanoscale Biophysics Group, FOM Institute AMOLF, Science Park 104, 1098 Amsterdam, The Netherlands; 3 Vrije Universiteit Amsterdam, De Boelelaan 1105, 1081 Amsterdam, The Netherlands

**Keywords:** Lipid–protein interactions, Nanodiscs, Styrene–maleic acid, SMALP, SMA-resistant membrane (SRM), Lipid rafts

## Abstract

A promising tool in membrane research is the use of the styrene–maleic acid (SMA) copolymer to solubilize membranes in the form of nanodiscs. Since membranes are heterogeneous in composition, it is important to know whether SMA thereby has a preference for solubilization of either specific types of lipids or specific bilayer phases. Here, we investigated this by performing partial solubilization of model membranes and analyzing the lipid composition of the solubilized fraction. We found that SMA displays no significant lipid preference in homogeneous binary lipid mixtures in the fluid phase, even when using lipids that by themselves show very different solubilization kinetics. By contrast, in heterogeneous phase-separated bilayers, SMA was found to have a strong preference for solubilization of lipids in the fluid phase as compared to those in either a gel phase or a liquid-ordered phase. Together the results suggest that (1) SMA is a reliable tool to characterize native interactions between membrane constituents, (2) any solubilization preference of SMA is not due to properties of individual lipids but rather due to properties of the membrane or membrane domains in which these lipids reside and (3) exploiting SMA resistance rather than detergent resistance may be an attractive approach for the isolation of ordered domains from biological membranes.

**Electronic supplementary material:**

The online version of this article (doi:10.1007/s00249-016-1181-7) contains supplementary material, which is available to authorized users.

## Introduction

In recent years, the styrene–maleic acid (SMA) copolymer has evolved as an important tool for isolation and characterization of membrane proteins [for review, see (Dörr et al. 2016)]. SMA has been shown to solubilize biological membranes in the form of nanodiscs allowing the isolation of membrane proteins directly from their native environment without the need for detergent (Long et al. 2013; Gulati et al. 2014; Jamshad et al. 2015; Prabudiansyah et al. 2015; Swainsbury et al. 2014; Dörr et al. 2014). The small size of these so-called “native nanodiscs” enables their characterization by a variety of biophysical approaches (Swainsbury et al. 2014; Dörr et al. 2014, 2016; Orwick et al. 2012; Jamshad et al. 2014; Orwick-Rydmark et al. 2012; Vargas et al. 2015) Furthermore, the presumed preservation of the annular lipid environment helps to maintain the stability of the embedded proteins and thereby allows the use of SMA as a convenient tool to study preferential lipid–protein interactions, simply by analyzing the lipid composition of purified protein-containing nanodiscs and comparing it with that of the native membrane (Swainsbury et al. 2014; Dörr et al. 2014; Prabudiansyah et al. 2015).

For unambiguous analysis of preferential lipid–protein interactions using SMA, it is however of crucial importance to know whether or not SMA by itself exhibits any lipid preference during solubilization. This can be conveniently investigated by employing synthetic model membrane systems, which allow highly systematic variation of lipid composition. Solubilization of model membranes by SMA results in the formation of styrene–maleic acid lipid particles (SMALPs), which have similar sizes and properties as membrane protein-containing (native) nanodiscs (Dörr et al. 2016). Using such model systems, it has been shown that the interaction of SMA with membranes strongly depends on lipid composition, with the kinetics of solubilization being modulated by, e.g., surface charge, lipid packing and lipid chain length (Scheidelaar et al. 2015). This would suggest that SMA might exhibit a lipid preference toward solubilization. However, experiments in which model membranes of an *Escherichia coli* total lipid extract were partially solubilized showed that the SMA-solubilized fraction exhibits no significant enrichment in specific lipid species (Scheidelaar et al. 2015). Together these results suggest that SMA is promiscuous and that solubilization is determined by overall properties of the membrane rather than by properties of individual lipids. This was supported by a recent study using ^31^P NMR (Cuevas Arenas et al. 2016).

So far, the lipid mixtures that have been used to study preferential solubilization by SMA represent only a few selected homogeneous lipid mixtures in the fluid phase, and no systematic studies have been reported yet on a possible lipid preference of SMA. Also, whether SMA exhibits any preference in heterogeneous membranes that exhibit domain formation and that arguably are biologically more relevant than homogeneous fluid bilayers has not been investigated.

To obtain insight into these matters, we here set out to investigate to what extent preferential solubilization of lipids by SMA occurs in simple binary lipid systems forming a single homogeneously mixed fluid phase and in heterogeneous phase-separated membranes exhibiting coexistence of a fluid phase with either gel or liquid-ordered phase. In order to maximize our “window” for monitoring any potential preferences of the polymer, the following strategy was employed. First, combinations of lipids were selected that on their own would have very different SMA solubilization kinetics. Second, to achieve partial solubilization short incubation times of 1 h were used thereby avoiding full equilibration of the system. This required adjustment of the concentration of SMA for each system individually in order to obtain sufficient material for reliable analysis. Third, multilamellar vesicles (MLVs) were chosen as the lipid system, which has the following advantages: (1) MLVs provide a large accessible surface area, which diminishes the chance of “all or nothing” effects that may occur for small vesicles, i.e., full solubilization of some membranes and no solubilization of others, (2) by employing these larger structures any potential curvature effects are avoided, and (3) the use of these larger structures facilitates the separation of solubilized and non-solubilized material by centrifugation.

The results show that SMA is indeed highly promiscuous with respect to solubilization of lipid species in homogeneous fluid bilayers, but that there is a clear preference for solubilization of the fluid phase in phase-separated bilayers with either a gel phase or a fluid phase. We will discuss the implications of these findings regarding the general applicability of SMA as a tool to determine preferential lipid–protein interactions. We will also discuss the use of SMA for the isolation of SMA-resistant membranes (SRMs) as an alternative to conventionally studied detergent-resistant membranes (DRMs).

## Materials and methods

### Materials

All lipids were purchased from Avanti Polar Lipids (Alabaster, AL). The used lipids were 1,2-dioleoyl-*sn*-glycero-3-phosphocholine (di-18:1 PC); 1,2-dioleoyl-*sn*-glycero-3-phospho(1′-rac-glycerol) (di-18:1 PG); 1,2-dioleoyl-*sn*-glycero-3-phosphoethanolamine (di-18:1 PE); 1,2-distearoyl-*sn*-glycero-3-phosphocholine (di-18:0 PC); 1,2-dimyristoleoyl-*sn*-glycero-3-phosphocholine (di-14:1 PC); 1,2-di-(9Z-hexadecenoyl)-*sn*-glycero-3-phosphocholine (di-16:1 PC); 1,2-di-(11Z-eicosenoyl)-*sn*-glycero-3-phosphocholine (di-20:1 PC); 1,2-dierucoyl-*sn*-glycero-3-phosphocholine (di-22:1 PC); 1-palmitoyl-2-oleoyl-sn-glycero-3-phosphocholine (16:0/18:1 PC); brain sphingomyelin (bSM); cholesterol; 23-(dipyrrometheneboron difluoride)-24-norcholesterol (Top-Fluor-cholesterol); 1,2-dioleoyl-*sn*-glycero-3-phosphoethanolamine-*N*-(lissamine rhodamine B sulfonyl) (rhodamine-PE).

Xiran 25010, a styrene–maleic anhydride copolymer with a molar ratio of styrene-to-maleic anhydride of 3:1 and an average molecular weight of 10 kDa, was a kind gift from Polyscope (Geleen, The Netherlands). Xiran 25010 (anhydride copolymer) was converted to the acid form by hydrolysis under base-catalytic conditions as detailed elsewhere (Scheidelaar et al. 2015). All other chemicals used were from Sigma Aldrich (St. Louis, MO).

### Preparation of multilamellar vesicles (MLVs)

Phospholipid stock solutions in chloroform/methanol (9:1 v/v) were mixed in predetermined ratios, and the solvent was removed under a stream of N_2_. The resulting lipid film was dried in a desiccator under vacuum for at least 1 h. MLVs were obtained by hydrating the lipid films with solubilization buffer (50 mM Tris–HCl, 150 mM NaCl, pH 8.0). The samples were then subjected to ten freeze–thaw cycles, each consisting of 3 min of freezing in liquid N_2_ (−196 °C) and 3 min of thawing in a water bath at 50 °C, well above the gel-to-fluid phase transition temperature (*T*_m_) of the lipids. For vesicles containing di-18:0 PC [*T*_m_ = 56 °C (Marsh 2013; Lewis et al. 1987)], the water bath was kept at 60 °C to ensure membrane fluidity.

### Turbidimetry experiments

The solubilization of MLVs was monitored by turbidimetry, using a Lambda 18 spectrophotometer (PerkinElmer, Waltham, MA) as described previously (Scheidelaar et al. 2015). Briefly, 700-µl aliquots of 0.5-mM dispersions of MLVs in solubilization buffer were transferred to a quartz cuvette and equilibrated at the desired temperature for 10 min. Next, different amounts of SMA were added as detailed in the legends of the corresponding figures, and the solubilization kinetics were followed at a fixed wavelength of 350 nm by monitoring the decrease of the apparent absorbance. Absorbance values were recorded every 0.4 s.

### Analysis and quantification of the lipid composition of solubilized fractions

#### Partial solubilization of vesicles

To achieve partial solubilization while still obtaining enough lipid material for further analysis, the SMA concentration was tuned for each lipid mixture individually. Accordingly, 700-μl aliquots of 0.5-mM dispersions of MLVs in solubilization buffer were supplemented with an amount of SMA that led to a decrease in apparent absorbance to approximately 50–60% after 1 h of incubation. Vesicles containing liquid-ordered domains required higher amounts of SMA for sufficient solubilization because of their higher solubilization resistance. Detailed incubation conditions for each sample are specified in the legends of the corresponding figures.

#### Lipid isolation

After 1 h of incubation with SMA, the samples were cooled down on ice and then transferred to a pre-chilled ultracentrifuge. The non-solubilized material was removed by centrifugation at 115,000×*g* for 1 h at 4 °C, and the supernatant, containing the solubilized lipid material, was collected. The lipids from the supernatant and from an aliquot of non-treated MLVs were extracted according to the method of Bligh and Dyer (Bligh and Dyer 1959) (see Supporting Information) prior to analysis.

#### Lipid analysis and quantification

The procedure for lipid analysis and quantification was selected depending on the lipid composition of the membrane. Lipids with different headgroups were separated by thin layer chromatography (TLC). Quantification was then achieved by densitometric analysis after copper staining (Dörr et al. 2014; Swainsbury et al. 2014). Phospholipids with the same headgroup but with unsaturated acyl chains of different length were separated by reverse-phase TLC. After iodine staining, each spot was scraped off, and the amount of phosphate was determined by the method of Rouser (Rouser et al. 1970). For phospholipids with the same headgroup but with acyl chains differing in degree of unsaturation, reverse-phase TLC did not provide sufficient separation. These samples were therefore quantified by gas chromatography after esterification of the fatty acids (de Smet et al. 2012; Dörr et al. 2014). For detailed experimental descriptions, see Supporting Information.

### Transmission electron microscopy

Size characterization of the SMALPs present in the supernatant fractions resulting from turbidimetry experiments was performed by transmission electron microscopy. To this end, copper grids were prepared following the carbon flotation technique. Briefly, samples were diluted with solubilization buffer to a lipid concentration of 0.5–1 mM, and small aliquots were adsorbed on carbon-coated mica. The mica was then transferred to a staining solution containing 2% (w/v) sodium silico tungstate, causing detachment of the carbon film. Subsequently, a copper grid was placed on top of the detached carbon that was recovered and dried under air flow. Images were taken under low dose conditions at a nominal magnification of 49,000 with a T12 electron microscope (FEI, Hillsboro, OR) at an operating voltage of 120 kV using an ORIUS SC1000 camera (Gatan, Inc., Pleasanton, CA). The average size of the SMALPs was estimated manually from 16 well-defined individual particles randomly located through the image based on their maximum diameter using Adobe Illustrator software (San Jose, CA). This procedure was used to avoid potential artifacts such as stain-induced particle aggregation or inhomogeneous particle staining (Zhang et al. 2011; Wan et al. 2011; Scheidelaar et al. 2015).

### Fluorescence imaging

Fluorescence microscopy imaging was performed at room temperature using a Nikon A1 confocal microscope (Tokyo, Japan) equipped with a Perfect Focus system. Supported lipid bilayers (SLBs) were prepared in a custom-built chamber following the vesicle fusion procedure (see Supporting Information). Solubilization of SLBs by SMA was assessed under a continuous flow of solubilizing agent solution. Images were taken before addition of SMA and after 5 min of incubation using a 100× oil immersion 1.49-NA objective (Nikon) under identical conditions of laser power and gain for all samples. Top-Fluor cholesterol and rhodamine-PE were imaged sequentially using a 488- and 561-nm laser, respectively, to avoid spectral cross-talk. The images were acquired with a resolution of 512 × 512 pixels (pixel size 0.41 × 0.41 μm).

Fluorescence intensities were quantified from intensity histograms using NIS Elements software (Nikon). Intensity values are expressed as an average of the intensities calculated from five different snapshots randomly picked from the planar bilayer. A representative video of the solubilization process can be found in the Supporting Information (Video S1).

## Results

### SMA does not display a preference for individual lipid species when solubilizing homogeneously mixed bilayers

It was previously demonstrated in model membrane systems that the lipid headgroup and acyl chain composition are important determinants for the kinetics of SMA solubilization (Scheidelaar et al. 2015). Here, we investigated whether two lipids that exhibit very different solubilization kinetics will be selectively solubilized by SMA when homogeneously mixed in a lipid bilayer. For this we selected mixtures of di-18:1 PC as “host” lipid with an equimolar amount of different “guest” lipids. The choice of these guest lipids was motivated by our previous observations (Scheidelaar et al. 2015) that (1) lipids with short chains are solubilized faster than lipids with longer acyl chains, most likely as a consequence of the lower number of van der Waals interactions between neighboring chains; (2) bilayers containing negatively charged lipids exhibit much slower solubilization kinetics than bilayers of zwitterionic lipids, presumably due to electrostatic repulsion by the negative charge of the polymer; (3) bilayers containing lipids in the gel phase, cone-shaped lipids or unsaturated lipids are solubilized more slowly than bilayers containing cylindrical lipids or saturated lipids in the fluid phase. These latter effects were ascribed to differences in the packing density of the acyl chains, with tighter packing hindering the insertion of the polymer and subsequent solubilization.

**Fig. 1:**
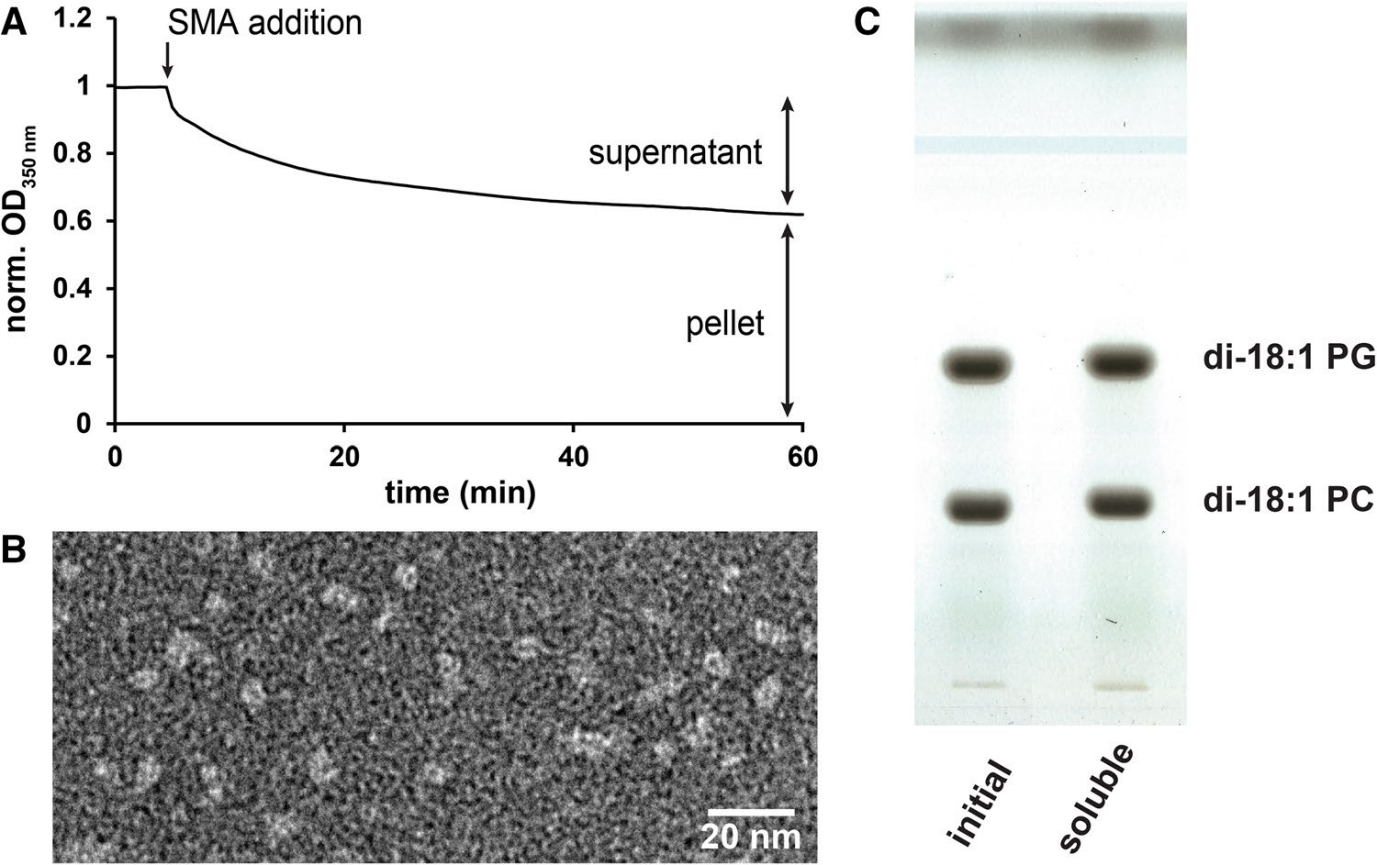
Partial solubilization of vesicles by SMA. **a** Kinetics of SMA solubilization of MLVs composed of an equimolar mixture of di-18:1 PC and di-18:1 PG (0.5 mM lipid, SMA-to-lipid mass ratio of 0.64) at 25 °C. Data are shown as normalized optical density at 350 nm. **b** Visualization of the SMALPs from the supernatant by negative-stain transmission electron microscopy. **c** Thin layer chromatography analysis of the lipid composition of lipids extracted from non-treated MLVs as well as the soluble fraction after partial solubilization by SMA

**Fig. 2:**
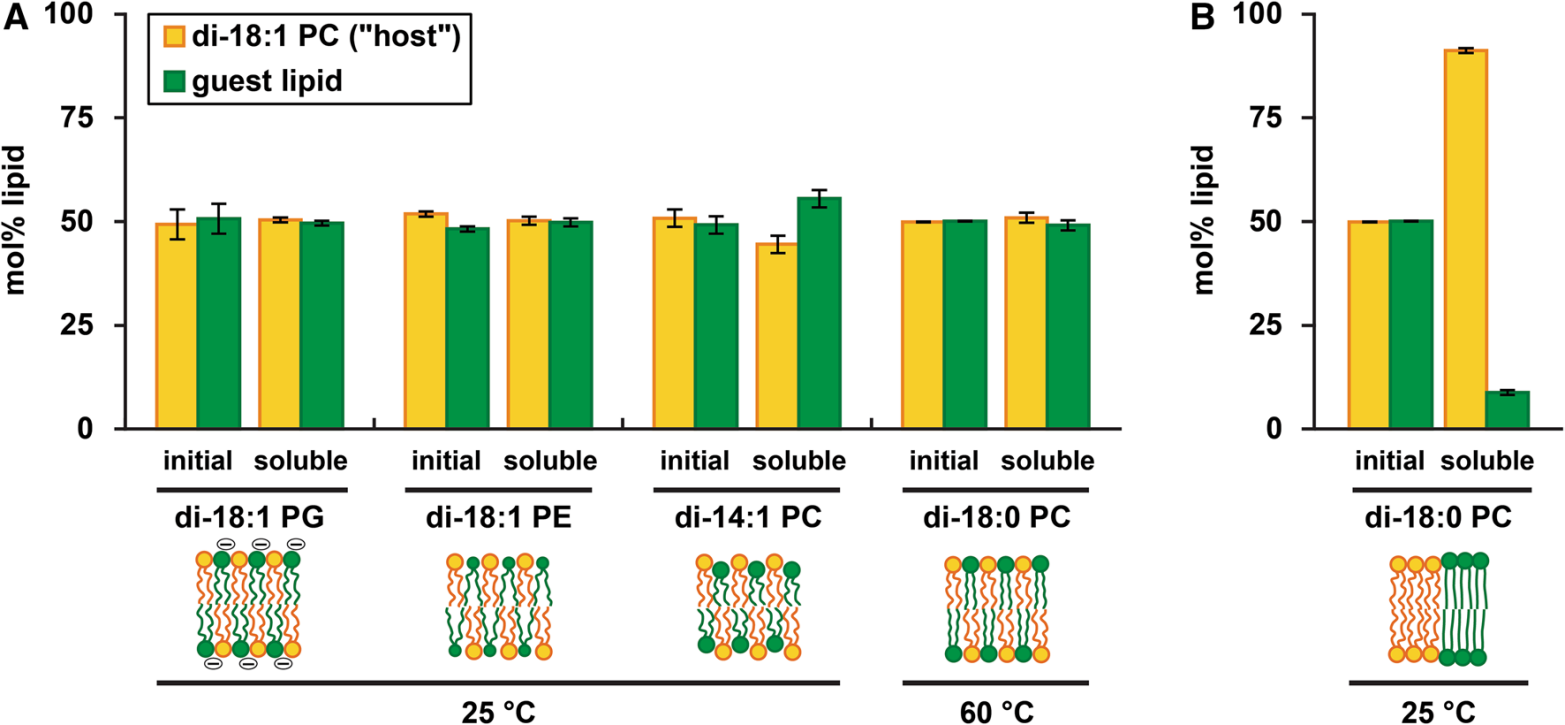
Solubilization preference of SMA in binary lipid systems with different properties assessed by lipid composition analysis after partial solubilization. **a** Equimolar mixtures of the zwitterionic unsaturated di-18:1 PC (“host”, *orange*) with different guest lipids (*green*) under conditions of phase homogeneity. From *left to right*: anionic di-18:1 PG, cone-shaped di-18:1 PE, short chain di-14:1 PC and saturated di-18:0 PC. Respective SMA-to-lipid mass ratios at 0.5 mM lipid were 0.64, 1.31, 0.27 and 0.13. Phase homogeneity for di-18:1 PC/di-18:0 PC was achieved by elevating the temperature to 60 °C, above *T*_m_ of di-18:0 PC (*T*_m_ = 56 °C) (Marsh 2013; Lewis et al. 1987). **b** Equimolar mixture of di-18:1 PC and di-18:0 PC under conditions of phase separation at 25 °C (SMA-to-lipid mass ratio 1.27). *Cartoons* show the schematic bilayer organization before addition of SMA. *Error bars* represent the standard deviation of three independent experiments

To obtain insights into a possible selectivity of SMA for certain lipids, an approach of partial solubilization of MLVs was used, as illustrated in Fig. 1a for a mixture of di-18:1 PC with the anionic lipid di-18:1 PG, which is known to form homogeneously mixed bilayers (Marsh 2013; Nibu et al. 1995). The soluble fraction, after incubation with SMA for 1 h, was subjected to electron microscopy (EM) imaging (Fig. 1b) and lipid composition analysis by TLC (Fig. 1c). The EM data (Fig. 1b) show a homogeneous distribution of particles of 6–8 nm size (Table 1), which is at the lower end of the range of commonly reported dimensions of around 10 nm [see, e.g., (Scheidelaar et al. 2015; Jamshad et al. 2014; Orwick et al. 2012)]. Lipid composition analysis of the solubilized fraction after SMA incubation revealed that PC and PG are present in a similar molar ratio as in the initial vesicles (Fig. 2a), indicating non-selective solubilization of both lipids. Considering the electrostatically unfavorable interaction of SMA with negatively charged lipids (Scheidelaar et al. 2015), this result is rather surprising and suggests that SMA does not perturb the bilayer homogeneity.

**Table 1:** Nanodisc size characterization based on analysis of EM data

Lipid mixture (1:1, M)	Incubation temperature (°C)	Size (nm)
di-18:1 PC/di-18:1 PG	25	6–8
di-18:1 PC/di-18:1 PE	25	8–10
di-18:1 PC/di-14:1 PC	25	6–8
di-18:1 PC/di-18:0 PC	60	8–10
di-18:1 PC/di-18:0 PC	25	6–8

Similar experiments were performed with other homogeneous lipid mixtures in the fluid phase. The results are summarized in Fig. 2a and Table 1, while original solubilization traces and representative EM micrographs can be found in Figure S1. When di-18:1 PC was mixed with the cone-shaped lipid di-18:1 PE, again it was found that SMA does not show a lipid preference (Fig. 2a), despite the slower kinetics of solubilization of PC/PE as compared to pure PC bilayers (Scheidelaar et al. 2015). This non-selective solubilization is in line with results from a recent study of a very similar lipid system (Cuevas Arenas et al. 2016). A different result was obtained for mixtures of lipids with varying acyl chain length, where a small preference was observed for solubilization of di-14:1 PC over di-18:1 PC (Fig. 2a). To elucidate whether this preference might be related to hydrophobic mismatch effects, we also tested mixtures in which di-14:1 PC was kept as the shorter lipid, while the length difference between the lipid components was either increased or decreased (Figure S2A). In all cases, the results showed a similar small preference for di-14:1 PC, suggesting that this is an artifact related to a particular feature of di-14:1 PC, perhaps being more easily extracted from the membrane because of its short unsaturated acyl chains. This hypothesis is supported by the results obtained from partial solubilization of di-18:1 PC/di-22:1 PC membranes (Figure S2B), where the solubilized fraction has a similar lipid composition as the initial vesicles. Finally, a mixture of di-18:1 PC with di-18:0 PC was tested under conditions where both lipids were in the fluid phase. This was achieved by raising the incubation temperature to 60 °C, above the gel-to-liquid crystalline phase transition temperature of di-18:0 PC. Here again no preference for either lipid species was observed (Fig. 2a).

For all the solubilized fractions corresponding to Fig. 2, the formed SMALPs appeared to have a rather similar size in the range of 6–10 nm as visualized by EM imaging (Fig. 1b, Figure S1) and as quantified in Table 1. Previously it was reported that the use of relatively low SMA concentrations might result in the formation of larger particles (Vargas et al. 2015; Zhang et al. 2015). However, in our case the different populations of SMALPs were found to be fairly small with a relatively uniform size distribution, despite conditions of relatively low SMA concentrations (SMA-to-lipid mass ratio of 0.3–1.3). Importantly, similar particle sizes were found under conditions of using a higher SMA-to-lipid ratio, longer incubation times and higher lipid concentrations, which allowed characterization of the particles by both EM and dynamic light scattering (Figure S2, Table S1). Together these data support the validity of our partial solubilization approach.

Overall, the data show that SMA is highly promiscuous with respect to solubilization of lipid species when these are present as homogeneously mixed bilayers in the fluid phase. Whether preferences of SMA solubilization do occur in bilayers with a heterogeneous lipid distribution was investigated next.

### SMA preferentially solubilizes the fluid phase under conditions where gel phase and fluid phase coexist

A heterogeneous lipid bilayer can easily be obtained in mixtures of lipids with unsaturated (low *T*_m_) and saturated (high *T*_m_) acyl chains by lowering the temperature well below T_m_ of the saturated lipid. For instance, in the above-described equimolar mixture of di-18:1 PC and di-18:0, lowering the temperature to 25 °C promotes a situation where gel and fluid (liquid–crystalline) phases coexist (Marsh 2013). Under these conditions, SMA shows a strong preference toward solubilizing the fluid phase, which is mainly constituted by di-18:1 PC (Fig. 2b). This result is in accordance with the much faster solubilization kinetics of lipids in the fluid phase as compared to lipids in the gel phase (Scheidelaar et al. 2015; Cuevas Arenas et al. 2016). For bilayers exhibiting phase coexistence, the lipid preferences of SMA under conditions of partial solubilization thus do appear to reflect the differences in solubilization kinetics between the lipids in their respective phases.

### SMA preferentially solubilizes the fluid liquid-disordered matrix upon incubation with membranes containing liquid-ordered domains

The resistance of gel-phase lipids against solubilization by SMA raises the question whether this is a general phenomenon for phases in which the lipids exhibit a high degree of order. This was first tested by adding SMA to a binary mixture of brain sphingomyelin (bSM) and cholesterol that forms a liquid-ordered (L_o_) phase (Sankaram and Thompson 1990; de Almeida et al. 2003). Addition of an amount of SMA that is generally sufficient to rapidly solubilizes homogeneous bilayers in the fluid phase (SMA-to-lipid mass ratio of 3.5) did not lead to any decrease in apparent absorbance after 1 h for this system (Figure S4), and neither did increasing the SMA concentration or prolonging incubation times (data not shown), indicating a very poor solubilization efficiency of SMA for lipids in the L_o_ phase. These results resemble those reported for the non-ionic detergent Triton X-100 (TX-100), for which the L_o_ phase shows a well-described detergent resistance [see, e.g., (El Kirat and Morandat 2007; Veiga et al. 2001; Rinia et al. 2001; Patra et al. 1999; Sot et al. 2002; London and Brown 2000)].

**Fig. 3:**
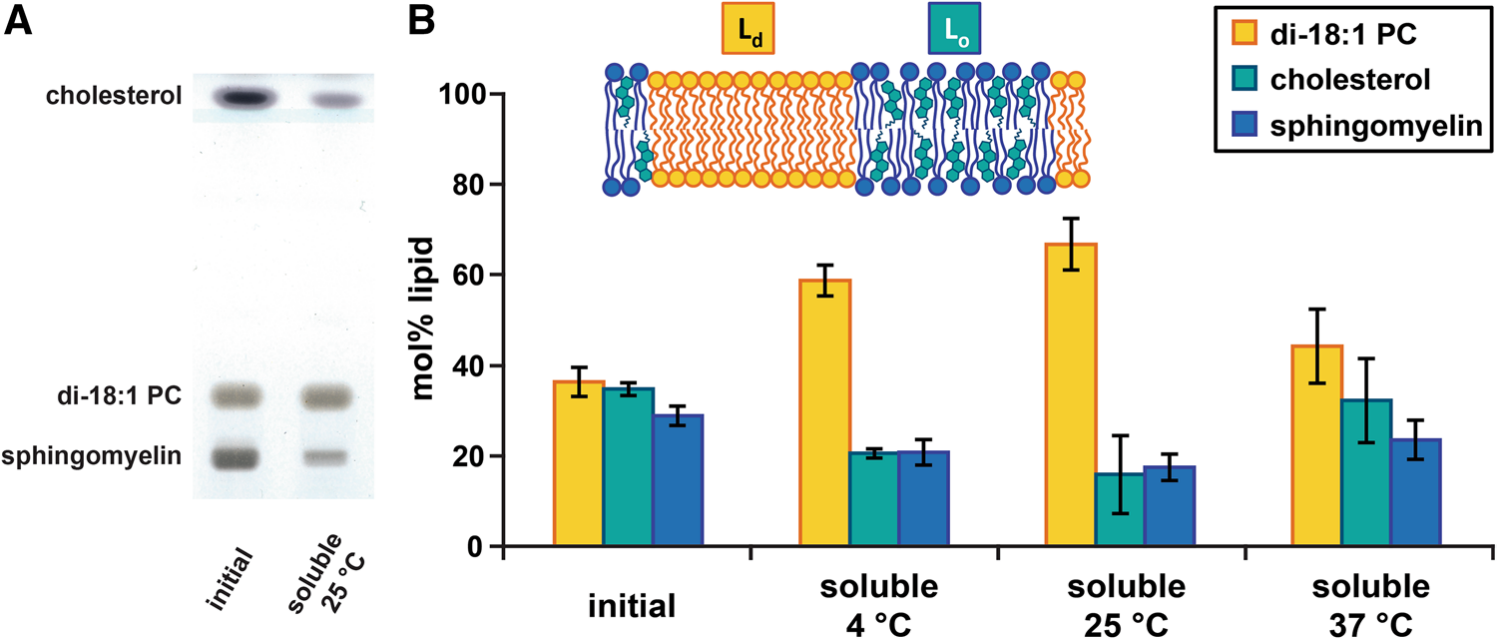
Lipid composition analysis after partial solubilization of MLVs composed of an equimolar ternary lipid mixture of di-18:1 PC, bSM and cholesterol by SMA. The inset shows a simplified schematic cartoon representation. **a** TLC plate with lipids extracted from non-treated vesicles and from the soluble fraction after incubation with SMA at 25 °C. **b** Quantification of lipid composition shown as mol% lipid (color coding consistent with cartoon) for non-treated vesicles as well as the solubilized fractions after the incubation with SMA (0.5 mM lipid, SMA-to-lipid mass ratio of 3.1) at different temperatures. *Error bars* represent the standard deviation of three independent experiments

The solubilization potential of SMA was further investigated in an equimolar ternary lipid mixture of di-18:1 PC, bSM and cholesterol. Over a wide temperature range, bilayers of such composition exhibit phase separation containing L_o_ domains enriched in sphingomyelin and cholesterol that coexist with a fluid liquid-disordered (L_d_) matrix enriched in di-18:1 PC (Veatch and Keller 2003; Marsh 2013; de Almeida et al. 2003). As shown from the TLC results in Fig. 3a and as quantified in Fig. 3b, the lipid material solubilized from these membranes after incubation with SMA at 25 °C is clearly enriched in di-18:1 PC, while it is depleted in bSM and cholesterol in approximately equimolar amounts. At 4 °C, the SMA-solubilized fraction has a rather similar lipid composition as at 25 °C, while at 37 °C the solubilized fraction resembles the non-treated case more closely (Fig. 3b). These results are consistent with a preferential solubilization of the L_d_ phase over the L_o_ phase by SMA at lower temperatures, which may be ascribed to tight packing and preferential SM–cholesterol interactions that cause co-segregation from the fluid phase (Veiga et al. 2001; Sankaram and Thompson 1990; Patra et al. 1999; Veatch and Keller 2003). Indeed, in the absence of cholesterol, an equimolar mixture of bSM and di-18:1 PC was found to be solubilized in equimolar amounts of both lipids (Figure S5), demonstrating the large effect of cholesterol on lipid organization. At 37 °C, both cholesterol and bSM are more readily solubilized, likely to be related to the beginning of a gradual liquid ordered-to-fluid phase transition (Lichtenberg et al. 2005; McMullen et al. 2004). Based on our findings of non-preferential SMA solubilization in homogeneous bilayers (Fig. 2a), it is likely that also this phase-separating ternary lipid mixture will be solubilized without a (strong) preference in case it exists in a homogeneous fluid phase. Similar results showing a high predisposition of the SMA copolymer to solubilize the L_d_ phase over the L_o_ phase were obtained for a phase-separating ternary lipid mixture of 16:0/18:1 PC, bSM and cholesterol (Figure S6).

**Fig. 4:**
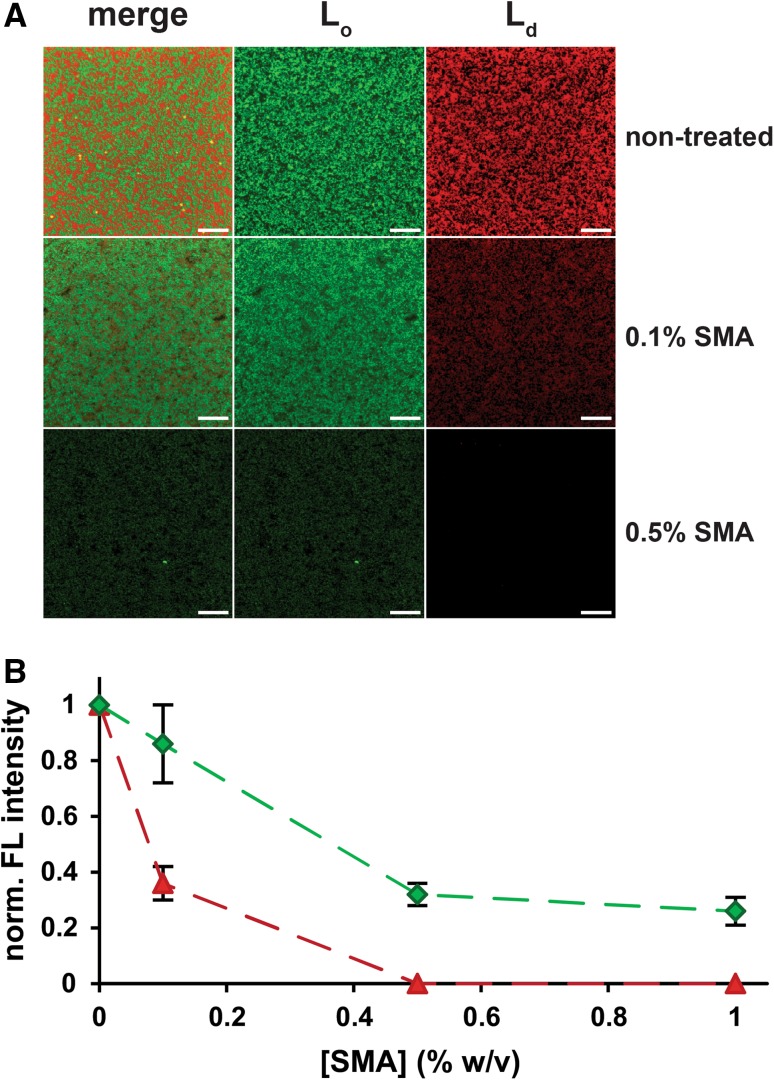
Preferential solubilization in supported lipid bilayers composed of an equimolar ternary lipid mixture of di-18:1 PC, bSM and cholesterol. **a** Fluorescence microscopy images are shown as merged (*left column*) and single channels (*green* top-fluor-cholesterol, middle column; red: rhodamine-PE, *right column*) for non-treated samples and after incubation times of 5 min with different amounts of SMA in solubilization buffer. The *scale bars* correspond to 10 µm. **b** Quantification of the fluorescence intensity in the images from (**a**). *Dashed lines* are depicted to guide the eye. All experiments were performed at room temperature. *Error bars* represent the standard deviation of the fluorescence intensity of five snapshots randomly picked from the planar bilayer

To gain more insight into the mode of action of SMA in phase-separated bilayers, we performed additional experiments where the solubilization of di-18:1 PC/bSM/cholesterol membranes was monitored using fluorescence microscopy. For these experiments we used supported lipid bilayers (SLBs) that were supplemented with a small amount of lipid-derived fluorescent dyes that partition selectively into the L_o_ or L_d_ phase (Klymchenko and Kreder 2014). At room temperature, the SLBs showed a clear phase separation, with L_o_ domains varying in size from 0.1–2 μm (Fig. 4a). After 5 min of incubation with 0.1% (w/v) of SMA, the fluorescence intensity of the L_d_ probe dropped by more than 50%, while the fluorescence emitted by the L_o_ probe decreased only marginally (Fig. 4b). When the SMA concentration was increased to 0.5% (w/v) only background levels of L_d_ fluorescence could be detected, while the L_o_ fluorescence was still at approximately 40% of the initial intensity. Importantly, no further decrease in L_o_ fluorescence was observed when the SMA concentration was further increased to 1% (w/v) (Fig. 4b, see also Video S1) or when the sample was allowed to further incubate with the SMA solution for several hours (data not shown). Thus, the L_d_ phase enriched in di-18:1 PC is efficiently solubilized by SMA, while the L_o_ domains show a high resistance against solubilization, which is in agreement with the experiments performed with vesicles at the same temperature.

## Discussion

The experiments described in this study reveal new insights into the process of membrane solubilization by SMA and how it depends on physicochemical properties of individual lipids and those of the membrane or membrane domains they reside in. Here, we will discuss our findings and the implications for the use of SMA as a tool to (1) study lipid–protein interactions and (2) isolate ordered domains from biological or model membranes.

### SMA as a tool to study lipid–protein interactions

To obtain insight into whether SMA by itself has any preference for solubilization of specific lipids, we performed partial solubilization experiments on binary lipid mixtures. The results strongly suggest that under conditions of phase homogeneity there is no significant preference of SMA to solubilize any glycerophospholipid species. This is irrespective of differences in solubilization kinetics of the individual lipids upon changing properties such as headgroup charge, lipid shape, acyl chain saturation or acyl chain length. A potential exception is di-14:1 PC, which was found to be incorporated into SMALPs with a slight preference, probably due to its short and unsaturated acyl chains. Importantly, the observed full promiscuity of SMA in solubilizing homogeneous fluid bilayers suggests that SMA by itself does not perturb the bilayer homogeneity. Thus, our findings support the validity of the use of SMA to study preferential lipid–protein interactions of membrane proteins that are extracted from biological membranes (Swainsbury et al. 2014; Dörr et al. 2014; Prabudiansyah et al. 2015). Here, a snapshot view of the interplay of lipids and proteins in biological membranes can be obtained, provided that the proteins reside in a fluid lipid environment.

However, biological membranes in general are heterogeneous and may contain domains that are more ordered (Simons and Ikonen 1997; Brown and Rose 1992; Schroeder et al. 1994). Our experiments with phase-separating lipid bilayers show that distinct solubilization preferences of SMA do arise under conditions of phase coexistence of a fluid phase with either a gel phase or a L_o_ phase. In both cases, the soluble fractions consisted almost exclusively of lipids that were in the fluid phase. For gel phases it was previously postulated that it is the tight packing of the chains that is responsible for the poor solubilization yield, because it increases the energetic barrier for SMA molecules to penetrate into the bilayer core (Scheidelaar et al. 2015; Cuevas Arenas et al. 2016). The same explanation would hold for the L_o_ phase, because it displays a similarly high degree of order of the acyl chains (Ipsen et al. 1987). Together these results demonstrate that lipid packing plays a major role in the resistance against solubilization by SMA. What are the implications of these results for the use of SMA for the investigation of preferential lipid–interactions for proteins that reside in either gel phase domains or liquid-ordered domains? Obviously, such proteins will not easily be solubilized into SMALPs. However, they may be isolated as insoluble domains instead, as will be further discussed below. Although analysis of the lipid environment in such a case will not provide a snapshot of the immediate lipid environment, it may nevertheless provide relevant information on the lipid composition of the domains in which the protein resides.

### SMA as a tool to isolate ordered domains

The clear preference of SMA to solubilize the fluid phase under conditions of phase coexistence holds promise for applications for the isolation of SMA-resistant membrane (SRM) domains. On the one hand, these could be applied to domains with a very high protein density, as was recently demonstrated by experiments in which SMA was used to prepare thylakoid membrane fractions that are enriched in specific photosystem complexes (Bell et al. 2015). On the other hand, they could involve approaches similar to those exploiting detergent resistance of certain membrane domains.

Resistance against detergent solubilization is a well-known phenomenon in membrane research, which has been used extensively to prepare DRMs from biological samples (Cerneus et al. 1993; Hanado et al. 1995; Brown and Rose 1992; Schroeder et al. 1994). In particular, TX-100 resistance at low temperatures has been exploited for the isolation of DRM fractions from mammalian plasma membranes. These DRM fractions have been associated to so-called “lipid rafts,” which are postulated to be specific membrane domains that are enriched in (glyco)sphingolipids, cholesterol and specific proteins and that have important roles in membrane function (Schroeder et al. 1994). The basis of their detergent resistance is ascribed to the ordered nature of the lipid chains in these domains (Simons and Ikonen 1997; Schroeder et al. 1994). Our results with model membranes suggest that SMA may be used in a similar way as conventional detergent to isolate highly ordered membrane domains in the form of SRMs.

This raises the question of how the two approaches to isolate ordered domains from biological membranes would compare. DRMs are unlikely to have the same composition as postulated natively occurring lipid rafts in the plasma membrane at physiological temperature (Heerklotz 2002; Lichtenberg et al. 2005). One reason for this is that conditions for DRM isolation usually include low temperatures, which will promote phase separation and thereby may cause further deviation from the composition of lipid rafts as they may occur at physiological temperature. Furthermore, by partitioning into the membrane, TX-100 shifts the thermodynamic equilibrium of phase separation (Heerklotz 2002) and thus likely affects the composition of lipid rafts that are isolated in DRMs. Alternatively, TX-100 has been postulated to increase the sizes of these domains (Pathak and London 2011).

It is not yet known to what extent this also holds for SMA. However, there is evidence that suggests that SMA may be less perturbing than detergent. It has been classified as an extraordinarily mild solubilizing agent (Vargas et al. 2015; Cuevas Arenas et al. 2016) having a very low free energy cost for solubilization of lipids from membranes into SMALPs. This is reflected by the native-like bilayer organization of the solubilized lipids (Jamshad et al. 2014). The results in the present study furthermore indicate that SMA is fully promiscuous in fluid bilayers, which suggests that SMA does not significantly perturb membrane homogeneity. Together, these results suggest that SMA could serve as an alternative for the isolation of highly ordered membrane domains that may have advantages over conventional methods using cold detergent solutions. However, whether indeed and to what extent SRMs isolated from biological membranes are superior to DRMs remains to be assessed.

Finally, an interesting novel possibility of this application of SMA may lie in the size of ordered domains existing in biological membranes. SMA may be capable of solubilizing very small membrane nanodomains in a conserved bilayer organization in case they are smaller than the average size of the SMALPs. This may for the first time make it possible to solubilize and characterize such small ordered domains directly from native membranes at physiological temperatures.

## Conclusions

In this study, we show that in fluid membranes SMA does not exhibit a preference for solubilization of specific lipids, which supports the validity of studying preferential lipid–protein interactions in SMA-bounded nanodiscs derived from either model or biological membranes. In phase-separated membranes, SMA has a strong preference for solubilization of the fluid phase, with the potential application of isolating ordered domains from biological membranes by exploiting their SMA resistance. Our initial data suggest that the use of SMA for these approaches may be an alternative to cold detergent solutions, which are commonly used for this purpose.

## Electronic supplementary material

Below is the link to the electronic supplementary material.

a representative video showing the solubilization of phase-separated SLBs upon SMA addition (Video S1) (AVI 37132 kb)

The supporting information includes turbidimetry data of all binary lipid mixtures shown in Figure 2 (Figure S1), a detailed size characterization of SMALPs by dynamic light scattering and EM (Figure S1, Figure S3 and Table S1), analysis of the lipid preference of SMA in di-14:1 PC-containing lipid mixtures (Figure S3), turbidimetry data on the SMA solubilization of membranes in an L_o_ phase (Figure S4), analysis of the lipid preference of SMA in a binary mixture of di-18:1 PC and bSM (Figure S5) and analysis of the lipid preference of SMA in 16:0/18:1-PC/bSM/cholesterol lipid mixtures (Figure S6) (DOCX 100551 kb)
