# Supplementary material for: Solubilization of lipids and lipid phases by the styrene–maleic acid copolymer
Source: Eur Biophys J. 2016 Nov 4;46(1):91–101. doi: 10.1007/s00249-016-1181-7 (PMC5209432; doi:10.1007/s00249-016-1181-7)
Supplement: Supplementary file 2 — The supporting information includes turbidimetry data of all binary lipid mixtures shown in Figure 2 (Figure S1), a detailed size characterization of SMALPs by dynamic light scattering and EM (Figure S1, Figure S3 and Table S1), analysis of the lipid preference of SMA in di-14:1 PC-containing lipid mixtures (Figure S3), turbidimetry data on the SMA solubilization of membranes in an Lo phase (Figure S4), analysis of the lipid preference of SMA in a binary mixture of di-18:1 PC and bSM (Figure S5) and analysis of the lipid preference of SMA in 16:0/18:1-PC/bSM/cholesterol lipid mixtures (Figure S6) (DOCX 100551 kb) [file 249_2016_1181_MOESM2_ESM.docx]

Supporting Information

Solubilization of lipids and lipid phases by the styrene–maleic acid copolymer

## Materials and Methods

### Dynamic light scattering

1-mL aliquots of 15 mM dispersions of MLVs were incubated with SMA (SMA-to-lipid mass ratio of approximately 1.8) overnight at the specified temperature (see Table S1). The non-solubilized material was pelleted down by spinning at 115,000 × *g* for 1 h at 4 °C and the supernatant, containing the solubilized lipid material, was collected. Excess SMA was removed from the supernatant using Amicon Ultra 0.5-mL centrifugal filters with a molecular weight cut-off of 30 kDa (Millipore, Darmstadt, Germany). The filtered solution was diluted to 1 mL with solubilization buffer aiming for a final lipid concentration of approximately 10 mM. Dynamic light scattering (DLS) analysis was performed on the dialyzed samples using a Zetasizer Nano ZS (Malvern Instruments, Worcestershire, UK). Samples were measured at least 12 times, each measurement being an average of 20 sub-runs of 15 s. Size–intensity distributions were generated using Zetasizer software Ver. 6.20 and 7.03 and analyzed using the multiple narrow distribution. Hydrodynamic diameters were calculated from the intensity distributions with the assumption that nanodiscs have a spherical shape. All samples showed a polydispersity index (PDI)<0.4.

### Transmission electron microscopy

Size characterization of the SMALPs present in the supernatant fractions resulting from DLS experiments was performed by transmission electron microscopy (TEM). To this end, samples were diluted with solubilization buffer to a lipid concentration of 0.5–1 mM and small aliquots were adsorbed on EM grids. The further procedure was as described in the main text.

### Lipid extraction

The lipids from solubilized fractions and from the initial vesicles were extracted according to the method by Bligh and Dyer (Bligh and Dyer 1959). Briefly, 700 μL of sample was mixed with 700 μL of CHCl_3_ and 1.61 mL of MeOH in a glass tube and the mixture was vortexed vigorously. Next, 700 μL of CHCl_3_ were added promoting phase separation and the organic phase was recollected. The aqueous phase was mixed once more with 700 μL of CHCl_3_ and vortexed. The organic phase was again recollected and added to the previous organic fraction obtained. The organic fraction containing the lipids was washed with a buffer solution (Tris 50 mM, EDTA 50 mM, pH 8.0) and mixed with 100 μL of isopropanol. The solvent was evaporated under a stream of N_2_ and the resulting lipid films were stored for further use.

### Lipid analysis and quantification

For thin layer chromatography, lipids extracted from the solubilized fraction and from the non-treated vesicles were dissolved in 100 μL chloroform/methanol (9:1 v/v) of which 20 μL and 10 μL, respectively, were applied to a high performance thin layer chromatography 10 x 10 cm Silica gel 60 plate (Macherey Nagel, Düren, Germany) using a Linomat 5 automatic TLC device (CAMAG, Muttenz, Germany). The lipids were separated in an ADC2 automatic development chamber (CAMAG), using chloroform/methanol/24% (v/v) ammonia/H_2_0 (68:28:3:1 v/v/v/v) as the mobile phase. Next, the plate was dried for 5 min under a stream of N_2_ and dipped in copper staining solution (10% w/v CuSO_4_, 8% v/v H_2_SO_4_ 98% w/v and 8% v/v H_3_PO_4_ 85% w/v). The spots were visualized by uniform heating at 130 °C for 15 min on a TLC plate heater (CAMAG) and subsequently quantified. The quantification was based on densitometry comparing the intensity of the lipid spots with a calibration curve on the same plate (Dörr et al. 2014; Swainsbury et al. 2014; Scheidelaar et al. 2015), using the Quantity One software (BioRad, Hercules, CA). In all cases, the amount of lipid in the samples was found to be in the linear range of the calibration curves.For reverse phase-TLC, the lipids extracted from the SMA-induced solubilized fraction and from the non-treated vesicles were dissolved in 100 μL chloroform/methanol (9:1 v/v) and 40 μL of each solution was applied manually to a silica gel C_18_ TLC plate (Millipore, Darmstadt, Germany). Next, the plate was developed in a TLC chamber, where the mobile phase consisted of methanol/dichloromethane/acetic acid (glacial) (80:20:1.5 v/v/v). After development, the plate was dried under a stream of N_2_ and immediately placed in an iodine tank to visualize the lipid spots. The spots were scraped off and the total phosphate content in each spot was quantified according to the method of Rouser (Rouser et al. 1970) as follows. Each sample was suspended in 300 μL of HClO_4_ (70 % w/v) and heated for 1.5 h at 170 °C until organic samples were completely converted to inorganic phosphate. Next, the reaction was cooled down by adding 1 mL of H_2_O followed by the addition of 0.4 mL of ascorbic acid (5% w/v) solution and 0.4 mL of ammonium molybdate (VI) tetrahydrate (1.25 % w/v) solution The samples were agitated and heated in a boiling water bath for 6 min and cooled at room temperature for at least 10 min. Absorbance of the samples was recorded at λ=797 nm, and the total phosphate amount was quantified from a calibration curve.

For gas chromatography, analysis was conducted after subjecting the phospholipids to an acid-catalyzed esterification (de Smet et al. 2012) as follows. The lipid films were suspended in 3 mL solution of 2.5 % w/v MeOH in H_2_SO_4_ (98% w/v) and heated for 2 h at 70°C. The reaction was stopped by adding 3 mL of H_2_O and the methylated fatty acids were extracted with 3 mL of hexane. The extraction was repeated and the organic fractions were combined. The organic fraction was washed twice with H_2_O after which 100 uL of isopropanol was added. The solvent was then removed under a stream of N_2_ stream and the resulting methylated fatty acid films were redissolved in 90 μL of hexane and saved for further analysis. Subsequently, the samples were analyzed using a TRACE GC Ultra (Interscience, Breda, The Netherlands) equipped with a Stabilwax polar column (Thermo Fisher scientific, Waltham, MA) with an internal diameter of 0.31 mm and a film thickness of 0.25 μm. The retention times of different fatty acid methyl esters were assigned by comparison with two standards: GLC 63b (Nu-Check Prep,Elysian, MN), and Bacterial Acid Methyl Ester Mix (Sigma-Aldrich, St.Louis, MO).

### Preparation of supported lipid bilayers

##### Substrate pretreatment

Glass slides were washed in 2 % (w/v) Hellmanex (VWR International, Chicago, IL) at 80 °C for 60 min, rinsed excessively with deionized water and then dried under a stream of N_2_. Cleaned slides were then etched for 8 min in a solution of 98 % w/v H_2_SO_4_ and 30 % w/v H_2_O_2_ (3:1 v/v). The slides were kept in MilliQ water and used immediately.

##### Preparation of supported lipid bilayers

Multilamellar vesicles (MLVs) were prepared in solubilization buffer from a mixture of di-18:1 PC, bSM and cholesterol in an equimolar ratio, supplemented with 0.01 mol% rhodamine-PE and 0.05 mol% Top-Fluor cholesterol (see Preparation of MLVs). Large unilamellar vesicles (LUVs) were then obtained by extrusion of the MLV dispersion 21 times through 100-nm polycarbonate membranes at 50 °C. Next, supported lipid bilayers (SLBs) were prepared by vesicle fusion as follows: a custom-built chamber (V = 100 µL) was assembled on top of the pretreated, hydrophilic glass slide. The chamber was then completely filled with a LUV dispersion (250 µM lipid) and equilibrated for 30 min. at room temperature. Subsequently, the unfused vesicles were removed by buffer flow and 2 chamber volumes of solubilization buffer containing different amounts of either SMA were flowed through the chamber at a flow speed of 50 µL/min using an oil-free pump.

*Monitoring the process of solubilization in real time*

The process of solubilization of supported bilayers (di-18:1 PC, bSM and cholesterol in an equimolar ratio, supplemented with 0.01 mol% rhodamine-PE and 0.05 mol% Top-Fluor cholesterol) was also monitored in real time for 5 minutes at a constant flow of 1% w/v SMA-containing buffer solution at room temperature.

## RESULTS

FIGURE S1: (Left) Kinetics of SMA solubilization of MLVs composed of equimolar lipid mixtures of 18:1-PC with di-18:1 PE (A), di-14:1 PC (B), di-18:0 PC (preparation at 60°C) (C) and di-18:0 PC (D). Solubilization was assessed at 25°C unless specified. Respective SMA-to-lipid mass ratios at 0.5 mM lipid were 1.31, 0.27, 0.13 and 1.27. Data are shown as normalized optical density (apparent absorbance) at 350 nm. (Right) Visualization of the nanodiscs from the corresponding supernatant fractions by negative-stain transmission electron microscopy.

FIGURE S2. (Left) Size distribution of soluble nanodiscs after removal of non-solubilized material as quantified by dynamic light scattering at 25 °C for equimolar mixtures of di-18:1 PC with di-18:1 PG (A), di-18:1 PE (B), di-14:1 PC (C), di-18:0 PC (preparation at 60°C) (D) and di-18:0 PC (E). The SMA-to-lipid mass ratio was kept constant at 1.8. Samples were prepared at 25 °C unless specified. (Right) Visualization of the SMALPs from the same sample by negative-stain transmission electron microscopy. In D, note the formation of stacks of discs in samples of di-18:1 PC and di-18:0 PC. Such “rouleaux” stacks have been observed before in or nanodiscs bounded by amphipathic proteins (Zhang et al. 2011; Wan et al. 2011) and were ascribed to an artifact resulting from specific interactions of the inorganic complexes of the staining solution with the positively charged choline headgroups.

Table S1 Nanodisc size characterization.

| Lipid mixture (1:1, molar) | Incubation temperature (°C) | Size EM (nm) | Size DLS (nm)* |
| --- | --- | --- | --- |
| di-18:1 PC/di-18:1 PG | 25 | 6–8 | 8.2 ± 0.9 |
| di-18:1 PC/di-18:1 PE | 25 | 5–8 | 8.8 ± 0.7 |
| di-18:1 PC/di-14:1 PC | 25 | 5–7 | 8.3 ± 1.0 |
| di-18:1 PC/di-18:0 PC | 60 | 7–9 | 8.1 ± 0.3 |
| di-18:1 PC/di-18:0 PC | 25 | 6–9 | 7.2 ± 0.7 |

*Errors reflect the accuracy of the positioning of the peak maximum within 12 consecutive measurements. Note however that the actual size distribution as estimated from the DLS intensity plots in Fig. S2 is several nm.


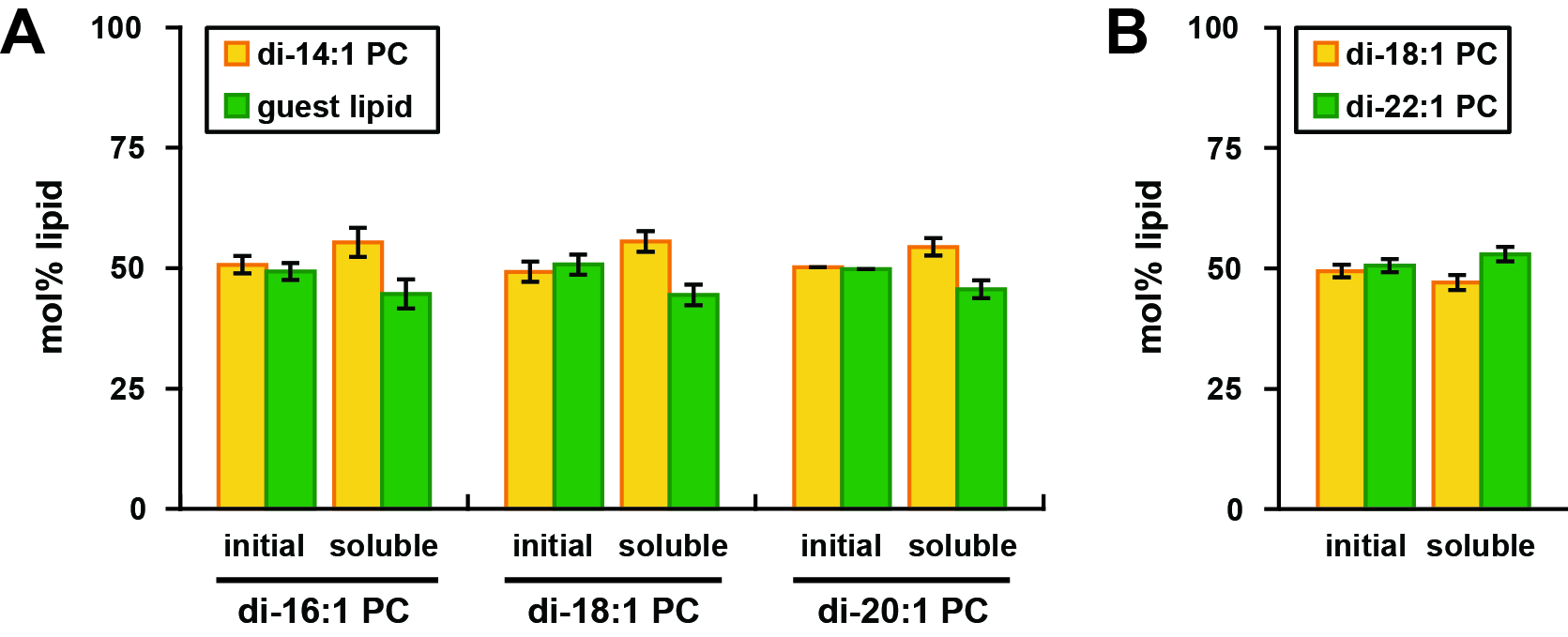


FIGURE S3. Solubilization preference of SMA in binary lipid systems with different properties assessed by lipid composition analysis after partial solubilization. Analysis was performed by reverse phase TLC (A) Equimolar mixtures of the zwitterionic unsaturated di-14:1 PC (“host”, orange) with different guest lipids (green) under conditions of phase homogeneity. From left to right: di-16:1 PC, di-18:1 PC and di-20:1 PC. Respective SMA-to-lipid mass ratios at 0.5 mM lipid were 0.28, 0.27 and 0.26. (B) Solubilization preference of SMA in equimolar mixture of di-18:1 PC and di-22:1: PC at SMA-to-lipid mass ratio of 0.24. Cartoons show the schematic bilayer organization before addition of SMA. Error bars represent the standard deviation of 3 independent experiments.


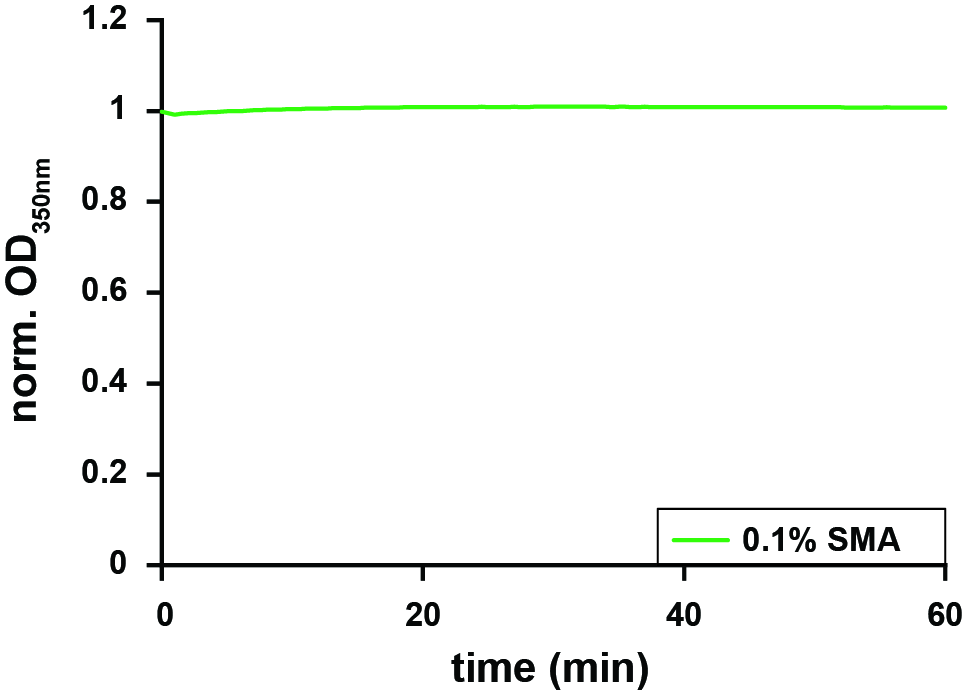


FIGURE S4. Inefficient SMA solubilization of membranes in the L_o_ phase. A turbidity trace is shown for MLVs composed of bSM and cholesterol (1:1, molar) that were incubated with SMA for 1 h (SMA-to-lipid mass ratio of 3.5).


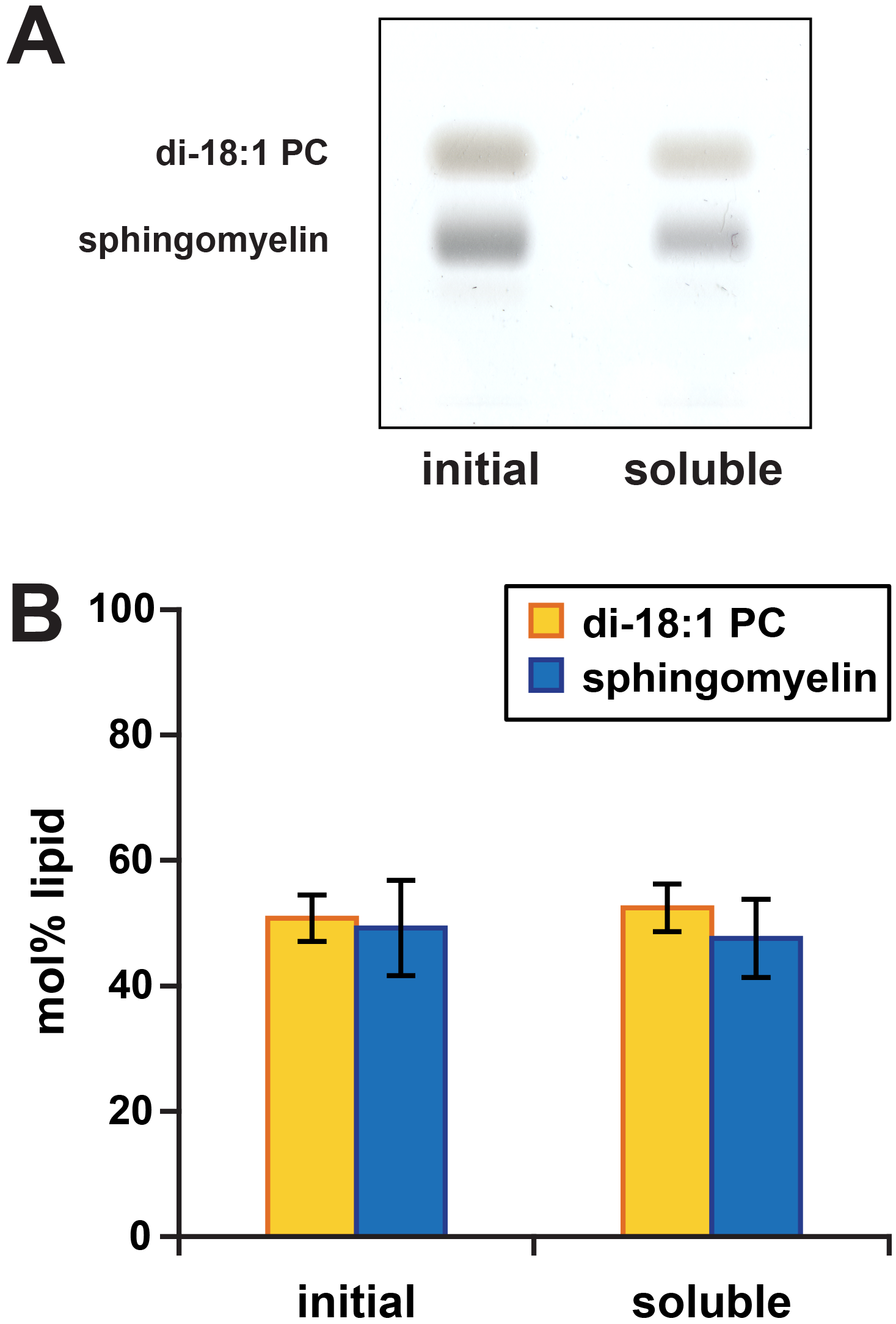


FIGURE S5. Lipid composition analysis after partial solubilization of MLVs composed of an equimolar binary lipid mixture of di-18:1 PC and brain sphingomyelin. (*A*) TLC plate with lipids extracted from initial vesicles and from the soluble fraction after incubation with SMA at 25 °C. (*B*) Quantification of lipid composition shown as mol% lipid for initial vesicles as well as the solubilized fractions after the incubation with SMA (0.5 mM lipid, SMA-to-lipid mass ratio of 2.6) at 25 °C. Error bars represent the standard deviation of 3 experiments.

FIGURE S6. Lipid composition analysis after partial solubilization of MLVs composed of an equimolar ternary lipid mixture of 16:0/18:1 PC, brain sphingomyelin and cholesterol by SMA. (*A*) TLC plate with lipids extracted from initial vesicles and from the soluble fraction after incubation with SMA at 4 °C, 25 °C and 37 °C. (*B*) Quantification of lipid composition shown as mol% lipid for initial vesicles as well as the solubilized fractions after the incubation with SMA at different temperatures (0.5 mM lipid, SMA-to-lipid mass ratio of 3.2). Error bars represent the standard deviation of 3 experiments.

VIDEO S1: Video showing the process of solubilization of a supported lipid bilayer of an equimolar mixture of di-18:1-PC/bSM/cholesterol, supplemented with the fluorescent lipids top-fluor-cholesterol (green, L_o_ domains) and rhodamine-PE (red, L_d_ domains) upon exposure to 1% (w/v) SMA in buffer at room temperature. The SLB was flushed continuously with SMA-containing buffer solution from the start of the video (begin of SMA incubation) until its end after 5 min (real time). L_d_ domains (red) disappear completely in time, whereas L_o_ domains remain intact, only showing a decrease in fluorescence intensity (see Figure 4 of the main text). For better representation the play speed of the video was accelerated threefold.

References

Bligh EG, Dyer WJ (1959) A rapid method of total lipid extraction and purification. Can. J. Biochem. Physiol. 37(8):911–917

de Smet, C. H., Vittone E, Scherer M, Houweling M, Liebisch G, Brouwers JF, de Kroon, A. I. P. M. (2012) The yeast acyltransferase Sct1p regulates fatty acid desaturation by competing with the desaturase Ole1p. Mol. Biol. Cell 23(7):1146–1156. doi: 10.1091/mbc.e11-07-0624

Dörr JM, Koorengevel MC, Schäfer M, Prokofyev AV, Scheidelaar S, van der Cruijsen, Elwin A. W., Dafforn TR, Baldus M, Killian JA (2014) Detergent-free isolation, characterization, and functional reconstitution of a tetrameric K + channel: The power of native nanodiscs. Proc. Natl. Acad. Sci. U. S. A. 111(52):18607–18612. doi: 10.1073/pnas.1416205112

Rouser G, Fleischer S, Yamamoto A (1970) Two dimensional thin layer chromatographic separation of polar lipids and determination of phospholipids by phosphorus analysis of spots. Lipids 5(5):494–496. doi: 10.1007/bf02531316

Scheidelaar S, Koorengevel MC, Dominguez Pardo J, Meeldijk JD, Breukink E, Killian JA (2015) Molecular Model for the Solubilization of Membranes into Nanodisks by Styrene Maleic Acid Copolymers. Biophys. J. 108(2):279–290. doi: 10.1016/j.bpj.2014.11.3464

Swainsbury DJK, Scheidelaar S, van Grondelle R, Killian JA, Jones MR (2014) Bacterial reaction centers purified with styrene maleic Acid copolymer retain native membrane functional properties and display enhanced stability. Angew. Chem. Int. Ed. Engl. 53(44):11803–11807. doi: 10.1002/anie.201406412

Wan C-PL, Chiu MH, Wu X, Lee SK, Prenner EJ, Weers PMM (2011) Apolipoprotein-induced conversion of phosphatidylcholine bilayer vesicles into nanodisks. Biochim. Biophys. Acta, Biomembr. 1808(3):606–613. doi: 10.1016/j.bbamem.2010.11.020

Zhang L, Song J, Cavigiolio G, Ishida BY, Zhang S, Kane JP, Weisgraber KH, Oda MN, Rye K-A, Pownall HJ, Ren G (2011) Morphology and structure of lipoproteins revealed by an optimized negative-staining protocol of electron microscopy. J. Lipid Res. 52(1):175–184. doi: 10.1194/jlr.D010959
